# Adolescent bariatric surgery—a survey of referring practitioners

**DOI:** 10.1007/s11845-024-03624-6

**Published:** 2024-03-08

**Authors:** Paul Cromwell, Therese McCarthy, Naomi Fearon, Helen Heneghan

**Affiliations:** Department of Surgery, St. Vincent’s University Hospital, Dublin, Ireland

**Keywords:** Bariatric surgery, Child and adolescent health, Obesity, Obesity management

## Abstract

**Background:**

Recent guidelines, supported by large, well-designed studies, suggest that bariatric surgery is a safe and effective treatment for adolescents living with severe obesity to improve health and psychosocial functioning. The aim of this study was to assess the opinions and referral practices of general practitioners (GPs) and paediatricians in Ireland.

**Methods:**

A cross-sectional survey was circulated online to practising paediatricians and GPs. The survey consisted of a short introduction about childhood obesity and 12 questions on adolescent bariatric surgery and obesity medications.

**Results:**

There were 45 unique responses to the survey from 22 GPs (48%), 8 paediatricians (17%), and 15 others. Most GPs (72%) would not consider referring an adolescent for bariatric surgery. Paediatricians were significantly more likely to refer (72% vs. 28%, *p* = 0.034). A minimum BMI of 40 kg/m^2^ was the most common response, which GPs (45%) and paediatricians (37.5%) suggested should be a pre-requisite for surgery. There was strong support for family psychological assessment and a reported deficit in the community support needed to manage obesity. GPs were more likely than paediatricians to respond that anti-obesity medications should be made available to adolescents, specifically liraglutide (45% vs. 25%), semaglutide (45% vs. 37.5%), and orlistat (22% vs. 0%).

**Discussion:**

There is a reluctance among GPs to refer adolescents with severe obesity for consideration of bariatric surgery. Concerns regarding the different obesity treatments held by medical professionals should be addressed through education and engagement and should be fundamental to the development of child and adolescent obesity services.

## Introduction

The current and long-term health of children with overweight and obesity is a serious global public health challenge. The rates of obesity are increasing in Ireland; it now ranks 10th among European countries with a rate of 9% among children aged 6–9 years old and is reported to be 13% among 20-year olds [1, 2]. In England, the National Child Measurement Programme reports that 20.1% of children aged 10–11 years have obesity [3, 4], comparable to 20.7% of children aged 6–11 years reported by the CDC in the USA [5]. Social determinants of health can drive obesity risks, such as poor access to healthy food, community stigma, family risk, and lower socioeconomic status. Within this environment, a child’s physiological, hormonal, genetic, and epigenetic factors can contribute to the development of obesity and related complications, including hypertension (HTN), type 2 diabetes (T2DM), non-alcoholic fatty liver disease (NAFLD), dyslipidaemia, obstructive sleep apnoea, and polycystic ovarian syndrome (PCOS) [6–9]. There is also thought to be an association with musculoskeletal disorders such as slipped capital femoral epiphysis and Blount’s disease [10].

Obesity stigma experienced by children and adolescents in the community and in a healthcare setting can prevent families from seeking out treatment, and those who do may find that options are limited and poorly resourced. Children and adolescents living with severe obesity in Ireland can be referred to a multidisciplinary service in Children’s Health Ireland (CHI) [11] that can provide lifestyle intervention, including family-based education on diet, exercise, healthy eating, psychological support, and access to medications. However, the mean BMI-SDS reduction is only − 0.17, and it is likely that only 10–15% of participants in this programme will see a clinically significant benefit from weight loss.

Internationally, there is substantial evidence that children do not grow into their weight, and therefore, a watchful waiting strategy is insufficient. The American Association of Paediatrics (AAP 2023) clinical practice guidelines recommend that treatment be offered early and immediately to children who have obesity [12]. Treatment recommendations from the AAP include motivational interviewing and longitudinal intensive family-based lifestyle advice and support, and in adolescents over 12 years, pharmacotherapy should be considered. For children and adolescents living with severe obesity, bariatric surgery should be considered. Bariatric surgery is also recommended in the National Model of Care for Obesity in Ireland [13]; however, it is currently unavailable for children and adolescents living with severe obesity. Prospective observational studies and randomised trials comparing adolescents undergoing bariatric surgery against controls given lifestyle advice [14–17] have demonstrated a durable reduction in weight [14–17], along with the feasibility and safety of surgery in this population [15, 18, 19]. There is evidence that adolescents who undergo bariatric surgery have higher rates of remission of T2DM [20], chronic kidney disease [21], and cardiovascular risk factors than reported in adults who undergo surgery.

There is variation in understanding of the role of surgical treatment for obesity in the adolescent population. A survey of healthcare professionals in the UK revealed that 66% of those surveyed felt that adolescents with a BMI of > 40 or > 35 with significant comorbidities should be offered bariatric surgery [22]. An older survey of US family physicians and paediatricians reported that 48% of respondents suggested that they ‘would never ever’ refer an adolescent for surgery [23]. As this is a developing need in Ireland, the aim of this survey was to investigate the current understanding and perception of general practitioners and consultant paediatricians towards adolescent bariatric surgery and, therefore, inform the development of future adolescent bariatric services, including the actions and education required for implementation, referral criteria, and follow-up services.

## Methods

A cross-sectional survey was circulated to fully qualified general practitioners and consultant paediatricians. Both groups were contacted directly through their publicly available professional e-mail addresses. Only currently registered practitioners in the Republic of Ireland were included. The survey results are reported in this article according to CROSS guidelines [24].

### Instrument

The survey was developed using the Survey Checklist Manifesto to mitigate the risk of bias [25]. It was generated online using Qualtrics XM software, copyright © [2023] Qualtrics. The survey comprised a short introduction followed by 12 questions. Nine questions had multiple-choice answers, and 3 were free text entries following a short question. The survey text is included as Supplementary Material A. All survey responses were recorded anonymously. This is a scoping survey with a convivence sample of physicians that will inform a larger, systematic assessment of the services required for the care of adolescents with severe obesity.

### Administration

Surveys were circulated in late 2022 to the target physicians along with an introductory covering statement about adolescent obesity. No follow-up e-mails were sent, and the survey was left open for several months after distribution. Results were tabulated and expressed as a percentage of respondents. The respondents were completely anonymous; the study investigators have no knowledge of who responded to the survey. Analysis was also conducted anonymously.

Descriptive and inferential analysis was performed using GraphPad Prism Version 9.3.0 for Mac (San Diego, California, USA, https://www.graphpad.com/). The descriptive analysis included frequency of response, median, range, and standard deviation. Inferential analysis was category vs. category: Chi-square analysis or Fischer’s exact test. Ethical approval was awarded by St. Vincent’s University Hospital Ethics Committee, and a dedicated question on the survey asked for consent for anonymised data to be used in the study. The survey was self-funded by the investigators, and no significant costs were encountered.

## Results

Forty-five healthcare professionals responded to the survey. Of the respondents, 48% were general practitioners, 17% were paediatricians, and 33% were other specialties (5 surgical consultants, 9 medical consultants, and 1 GP trainee). Sixty-four individuals accessed the survey, and 45 responses were given. All 45 respondents were unique visitors as identified by the Qualtrics online software. 22/246 (8.9%) of GPs who were approached responded to the survey and 8 of 290 (2.7%) of paediatricians responded.

**Fig. 1:**
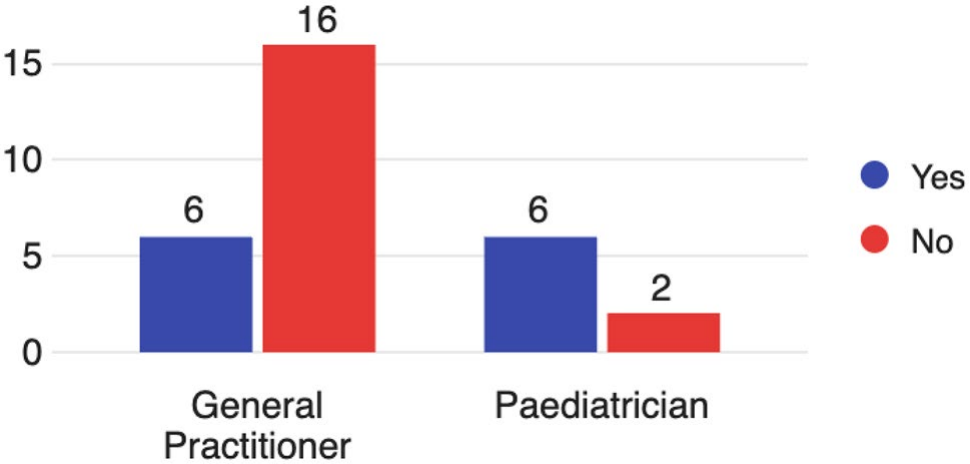
Would you consider referring an adolescent for weight-loss (bariatric) surgery? (*n* = 30 total, 22 GPs, 8 paediatricians)

Of all respondents, 46% would not refer an adolescent for bariatric surgery. Focusing on the target population of GPs and paediatricians (Fig. 1), 77% (*n* = 16) of GPs responded they would not consider referring an adolescent for bariatric surgery. In contrast to this, only 25% (*n* = 2) of paediatricians responded that they would not refer an adolescent. There was a significant difference identified between these two groups on statistical analysis (*p* = 0.034).

When asked for further comments about using bariatric surgery as a weight management tool in adolescents, responses included that bariatric surgery was ‘… a relatively high-risk procedure for what is mostly a societal problem’ and that we should ‘… spend more money on prevention/education… but accept in some situations it might be needed’. Other responses ranged from ‘Should only be for adults who have failed multiple attempts at exercise/diet measures for weight reduction’ to ‘it is important for a minority of adolescents and is absolutely part of the solution for some’.

### The minimum age that bariatric surgery should be considered

**Fig. 2:**
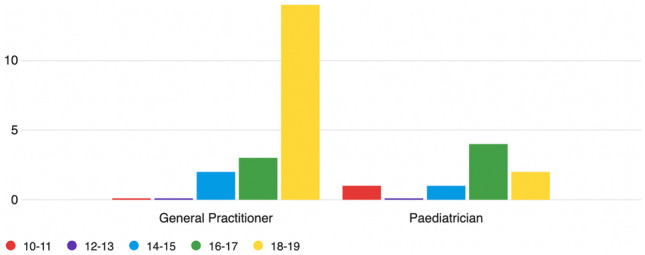
The minimum age at which bariatric surgery should be considered in adolescents. GPs versus paediatricians

Most respondents (47.6% of respondents) felt that 18 years old was the minimum age above which bariatric surgery should be offered. There was no difference between girls and boys in the minimum age recommended (49% vs. 48%). Figure 2 shows the minimum age at which bariatric surgery should be considered, grouped according to GPs versus paediatricians. Most (73.6%) GPs responded that 18 years old should be the minimum age versus 25% (*n* = 2) of paediatricians, half of whom felt that 16 years of age is an appropriate age to consider surgery.

### The minimum BMI where bariatric surgery should be considered

**Fig. 3:**
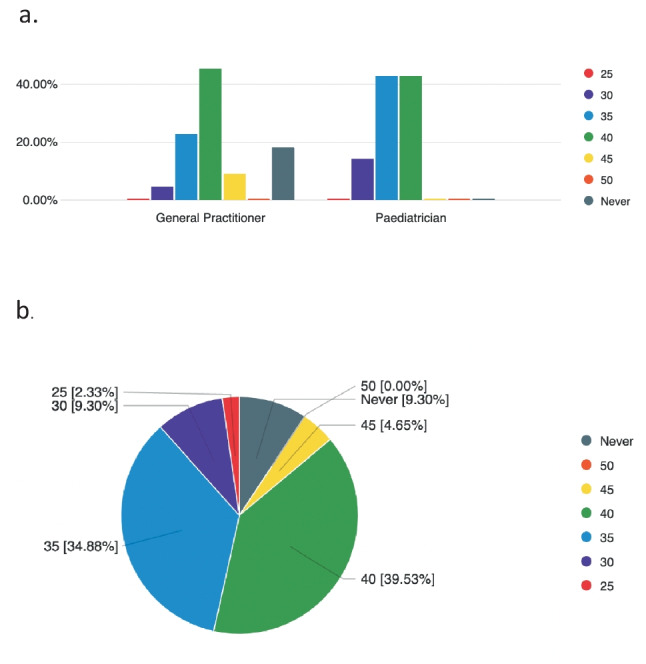
**a** Minimum BMI above which bariatric surgery could be considered in an adolescent. **b** All respondents had a minimum BMI above which bariatric surgery could be considered for an adolescent

Figure 3b demonstrates the range of responses regarding the minimum BMI at which bariatric surgery should be considered. Among GPs, 45% (*n* = 10) felt that a BMI ≥ 40 kg/m^2^, regardless of obesity complications, should be the minimum BMI at which bariatric surgery is considered. Around a third (37.5%) of paediatricians responded that a BMI ≥ 35 kg/m^2^ was sufficient, and an equal number responded that a BMI ≥ 40 kg/m^2^ would be appropriate. A number (18%) of GPs responded that they would not consider surgery appropriate at any BMI. Figure 3a shows the minimum BMI data in GPs and paediatricians.

### Minimum length of time attending weight management service and other supports

Approximately half (51%) of respondents felt that patients should attend an obesity service for at least 12 months before consideration for surgery. A small group (10%) of respondents felt 6 months was appropriate, and 38% (*n* = 18) suggested 18 months or more. Less than half (41%) of GPs responded that they would expect an adolescent to be in a weight management service for > 24 months.

There was broad agreement on the importance of psychological assessment and support. All paediatricians and nearly all (95.4%) GPs responded that an adolescent should have both individual counselling and family counselling before considering surgery. When asked about what further supports physicians expect to be in place, a dietician for the whole family was suggested, and the lack of such services currently was highlighted. Other prerequisites in addition to the obesity clinic were ‘supported counselling’ and ‘CBT and family education’.

General practitioners and paediatricians were divided on whether a child regularly missing school was an indication for surgery in an adolescent with a BMI ≥ 35. Half of paediatricians and 45% of GPs responded that missing school should be an indication for surgery.

### Longer-term follow-up

**Fig. 4:**
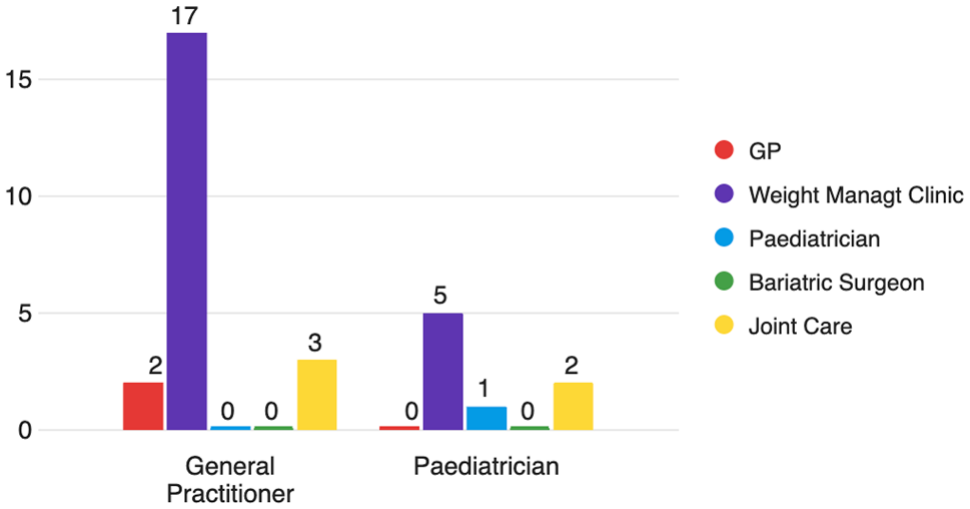
Who should follow-up with the patients long-term? Responses from GPs versus paediatricians

Figure 4 outlines who GPs and paediatricians believe should follow-up adolescent patients with obesity for long-term follow-up. The majority of GPs (77.2%, *n* = 17) and paediatricians (62%, *n* = 5) responded that a specialist obesity clinic should follow-up patients long-term. Less than 10% of GPs responded that they should carry out the follow-up. There was some support (22.7%) for a shared-care clinic that included GPs. Respondents did not expect a bariatric surgeon to carry out the long-term follow-up.

### Medications

**Fig. 5:**
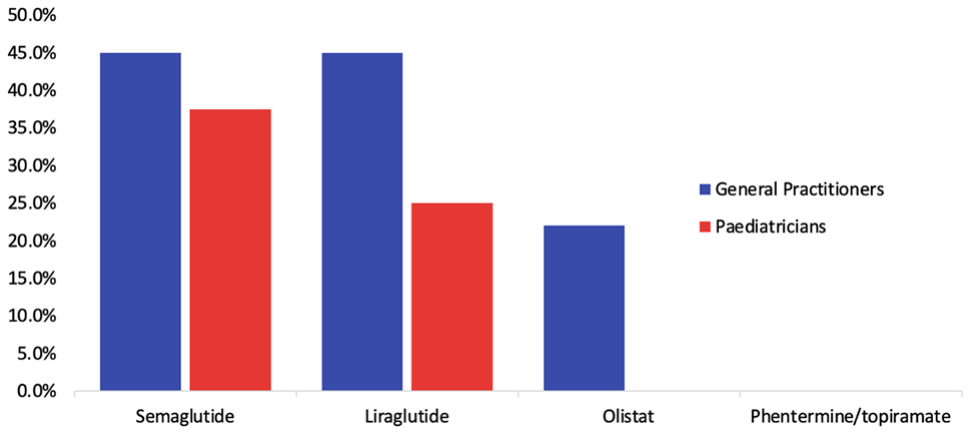
What medications should be made available to adolescents for weight management? GPs versus paediatricians

The respondents were asked what obesity medications should be made available to adolescents. The majority of GPs (55%, *n* = 12) and paediatricians (62.5%, *n* = 5) did not recommend any medication. However, GPs were more likely than paediatricians to respond that medications should be made available to adolescents for obesity management, specifically liraglutide (45% vs. 25%), semaglutide (45% vs. 37.5%), and orlistat (22% vs. 0%) (Fig. 5). None of the respondents felt that phentermine/topiramate should be available. Some additional answers included ‘… don’t know about the evidence of their use in this age group’, ‘unsure’, ‘never considered it’ and ‘antidepressants if indicated’.

## Discussion

This is the first study to present opinions on referring physicians to adolescent bariatric surgery services in Ireland. This survey revealed that most (72%) GPs would not consider referring an adolescent for obesity surgery. This is in contrast to paediatricians, most of whom (75%) responded that they would consider a referral. As this study was not performed in a focus group setting, the rationale behind this opinion is unknown. However, qualitative responses offer some explanation. Several respondents reported that they were not aware that bariatric surgery was available to adolescents with severe obesity. One respondent reported they had ‘… not yet come across a teenage patient whom I have considered…’ needing bariatric surgery. Others report that it should be reserved for adults who have not responded to other weight-loss strategies. It may be that there is a lack of understanding of obesity as a chronic and progressive disease and the mechanisms by which surgery and medical therapies work to treat the disease. There may be more awareness of the need for and benefit of these treatments among paediatricians.

The opinions in this survey are more conservative than those previously published in other countries, and this may reflect social and cultural differences; however, knowledge of the safety, efficacy, and perceived level of risk in adolescents was not specifically tested by this instrument. Neither was the bias and stigma associated with the treatment of obesity. The stigma surrounding obesity as a disease and the management of obesity is as prevalent in healthcare as it is in the general population [26]. Firm conclusions cannot be drawn from our data, but reluctance to refer patients may be a result of the pervasive ‘move more, eat less’ advice that is given by healthcare professionals worldwide.

Although 72% of GPs suggested that they would not refer an adolescent for bariatric surgery, most (81%) did respond with their view on the minimum BMI that they felt was appropriate for surgery in adolescents. This might reflect the absence of this service to date, but there is potential engagement going forward. Referral pathways for adolescents to obesity services could be examined closely with further study to investigate how best to educate and inform referring clinicians of treatments and pathways.

Nearly half (45%) of GPs that responded felt that semaglutide and/or liraglutide should be made available, and 38% of paediatricians responded that semaglutide should be available to adolescents for the treatment of obesity. Qualitative responses suggest that some GPs and paediatricians feel obesity medications are the responsibility of a tertiary weight management clinic. Furthermore, some respondents also reported that they have limited knowledge of the use and safety of medications in this age group. Therefore, it is unclear what level of knowledge exists for these relatively new medications in adolescents and whether further education on the topic would change opinions. Regarding other older medications, no paediatrician reported that orlistat or phentermine/topiramate should be made available to adolescents. This is perhaps due to their limited efficacy and known side effects; however, no further data were collected on the rationale for this decision. With recent high-level evidence available, this is an area that should be highlighted with education sessions through our clinical training programs and continuous professional development programs.

### Follow-up and supports

An important aspect of care identified by this survey is the lack of community resources and the high priority that referring practitioners put on perioperative support. Nearly all GPs and paediatricians responded that patients should have both individual counselling and family counselling before consideration surgery. This is in line with current guidelines [27], and it was clear that respondents felt there is not enough community-based support for obesity management currently.

Most GPs and paediatricians believe that adolescents should be followed up long-term in a specialist obesity clinic, with some respondents expressing support for joint care. Obesity is a life-long disease, and management is needed through the transition to adulthood; therefore, good communication and the development of management pathways between child and adult services are needed, along with the integration of community, secondary, and specialist care.

## Limitations

The checklist manifesto [25] was used to reduce bias. This was also a small study with convenience sampling through e-mail and a relatively high rate of non-responders. Although the survey captured valuable insights, respondents of this study will likely have been motivated to take part, and it is unclear whether the data represent the opinions of a majority of the practising GPs and paediatricians.

Before the implementation of an adolescent bariatric surgery service, national targeted focus-group-type research could be conducted with referring practitioners to clarify perceived barriers to referral and provide education to gain buy-in. Educational tools that are easy to access and readily available to physicians may be useful to support and encourage referrals to adolescent weight management services and could bridge knowledge gaps. Such education tools could include the latest evidence and guidelines regarding medical and surgical treatments for obesity, along with expected outcomes and follow-up guidance.
